# Criminal Abortion

**Published:** 1872-02

**Authors:** D. A. O’Donnell, W. L. Atlee

**Affiliations:** Committee; Committee


### CRIMINAL ABORTION.

We have received the report, in pamphlet form, of the Committee on Criminal Abortion to the American Medical Association, and very much regret that our space will only permit us to give place to the resolutions offered by the Committee. The report is an able document, and shows that the Committee were thoroughly impressed with the importance of their work. They have suggested, in our opinion, the only feasible way of arresting this wholesale murder. At the close of their report, the Committee offered the following resolutions:

*Resolved*, That we repudiate and denounce the conduct of abortionists, and that we will hold no intercourse with them either professionally or otherwise, and that we will, whenever an opportunity presents, guard and protect the public against the machinations of these characters by pointing out the physical and moral ruin which follows in their wake.

*Resolved*, That in the opinion of this Convention, it will be unlawful and unprofessional for any physician to induce abortion or premature labor, without the concurrent opinion of at least one respectable consulting physician, and then always with a view to the safety of the child, if that be possible.

*Resolved*, That we respectfully and earnestly suggest to private teachers, and professors in public institutions, the propriety of adopting, according to their judgment, the means best suited for preserving their pupils, and those who may hereafter come under their care, from the degrading crime of abortion.

*Resolved*, That we respectfully call the attention of the clergy of all denominations to the perverted views of morality entertained by a large class of females—aye, and men also—on this important question, and the ruin which has resulted, and continues to result daily, to the human family from such views.

*Resolved*, That we respectfully solicit the different medical societies, both State and local, to send delegates to the clergymen in their respective districts to request their aid in so important an undertaking.

*Resolved*, That it becomes the duty of every physician in the United States, of fair standing in his profession, to resort to every honorable and legal means in his power to crush out from among us this pest of society; and, in doing so, he but elevates himself and his profession to that eminence and moral standard for which God has designed it, and which an honorable and high-toned public sentiment must expect at the hands of its members.

D. A. O'Donnell, W. L. Atlee, } Committee.

